# The rice *white green leaf 2 gene* causes defects in chloroplast development and affects the plastid ribosomal protein S9

**DOI:** 10.1186/s12284-018-0233-2

**Published:** 2018-07-11

**Authors:** Zhennan Qiu, Dongdong Chen, Lei He, Sen Zhang, Zenan Yang, Yu Zhang, Zhongwei Wang, Deyong Ren, Qian Qian, Longbiao Guo, Li Zhu

**Affiliations:** 1 State Key Laboratory of Rice Biology, China National Rice Research Institute, Hangzhou, 310006 China; 2 Biotechnology Research Center, Chongqing Academy of Agricultural Sciences, Chongqing, 401329 China

**Keywords:** Plastid ribosomal protein, Albino phenotype, Chloroplast development, Rice

## Abstract

**Background:**

Plastid ribosomal proteins (PRPs) play important roles in the translation of key proteins involved in chloroplast development and photosynthesis. PRPs have been widely studied in many plant species; however, few studies have investigated their roles in rice.

**Result:**

In the present study, we used ethyl methane sulfonate mutagenesis and obtained a novel rice mutant called *white green leaf 2* (*wgl2*). The *wgl2* mutants exhibited an albino phenotype from germination through the three-leaf stage, and then gradually transitioned to green through the later developmental stages. Consistent with this albino phenotype, *wgl2* mutants had abnormal chloroplasts and lower levels of photosynthetic pigments. Map-based cloning and DNA sequencing analyses of *wgl2* revealed a single-nucleotide substitution (G to T) in the first exon of LOC_Os03g55930, which resulted in a substitution of glycine 92 to valine (G92 V). *WGL2* encodes a conserved ribosomal protein, which localizes to the chloroplast. Complementation and targeted deletion experiments confirmed that the point mutation in *WGL2* is responsible for the *wgl2* mutant phenotype. *WGL2* is preferentially expressed in the leaf, and mutating *WGL2* led to obvious changes in the expression of genes related to chlorophyll biosynthesis, photosynthesis, chloroplast development, and ribosome development compared with wild-type.

**Conclusions:**

*WGL2* encodes a conserved ribosomal protein, which localizes to the chloroplast. *WGL2* is essential for early chloroplast development in rice. These results facilitate research that will further uncover the molecular mechanism of chloroplast development.

**Electronic supplementary material:**

The online version of this article (10.1186/s12284-018-0233-2) contains supplementary material, which is available to authorized users.

## Background

The development of chloroplasts from proplastids involves plastid replication and activation of plastid DNA synthesis, chloroplast genetic system “build-up”, and synthesis and assembly of the photosynthetic apparatus. These steps are regulated by the coordinated expression of nuclear and plastid genes (Kusumi et al. 2010; Kusumi et al. 2011; Mullet 1993). Transcription of chloroplast genes depends on the nucleus-encoded RNA polymerase (NEP) and the plastid-encoded RNA polymerase (PEP) (He et al. 2018; Hedtke et al. 1997; Qiu et al. 2017; Shiina et al. 2005). Plastid and chloroplast development are affected by NEP and PEP throughout plant growth (Qiu et al. 2017). During chloroplast development, NEP preferentially transcribes plastid housekeeping genes, such as those encoding the PEP apparatus, rRNA, and tRNA, and the overall transcriptional and translational activities in the chloroplast dramatically increase (Hajdukiewicz et al. 1997). Later in chloroplast development, NEP becomes less important and chloroplast genes are transcribed by PEP (De Santis-MacIossek et al. 1999), and translated by the ribosomal complex (Pogson and Albrecht 2011).

Ribosomes are essential for protein synthesis, and in plants the ribosomes catalyze protein synthesis in the cytoplasm, plastids, and mitochondria (Zhang et al. 2016). Plastid ribosomal proteins (PRPs) play important roles in the build-up step of chloroplast differentiation. Plastid proteins are translated by the 70S ribosome, which consists of one small (30S) and one large (50S) ribosomal subunit; these have bacterial orthologs (Subramanian 1985). The small subunit consists of the 16S rRNA and 24 PRPs, including 12 proteins encoded by plastid genes and 12 encoded by nuclear genes (Yamaguchi and Subramanian 2003). The large subunit consists of three rRNAs (23S, 5S, and 4.5S) and 33 PRPs, including 8 proteins encoded by plastid genes and 25 encoded by nuclear genes (Yamaguchi et al. 2003). The essential and non-essential PRPs are partially conserved between bacteria and chloroplasts. Interestingly, some ribosomal proteins are not essential for the bacterial ribosome, but are essential for chloroplasts (Tiller and Bock 2014).

Since PRPs are crucial for plant growth and development, the lack of PRPs can affect protein synthesis and result in diverse phenotypes. Due to the lack of the plastid small subunit protein 17 (PRPS17), the maize *high chlorophyll fluorescent 60* (*hcf60*) mutant has a seedling-lethal phenotype (Schultes et al. 2000). The maize *lethal embryo 1* (*lem1*) mutant contains a mutation in the gene encoding a protein that is similar to the rice PRPS9, and *lem1* mutants have an early embryonic death phenotype (Ma and Dooner 2004). The tobacco mutant *plastid ribosomal protein S18* (*prps18*) displays defects in leaf development (Rogalski et al. 2006). The plastid-encoded proteins RPL33 and RPS15 are required under certain environmental conditions in tobacco, and improve ribosome biogenesis efficiency at elevated temperatures (Ehrnthaler et al. 2014; Rogalski et al. 2008). Plastid proteins play essential roles in embryo development, which has been shown with various plastid ribosomal protein mutants in Arabidopsis (*prps5*, *9*, *13*, *20* and *prpl1*, *4*, *6*, *10*, *13*, *18*, *19*, *21*, *22*, *27*, *28*, *31*, *32*, *35*, *36*) (Gong et al. 2013; Wang et al. 2017a). The Arabidopsis plastid ribosomal protein S5 (RPS5) plays a role in photosynthesis, plant development, and cold tolerance (Zhang et al. 2016). In rice, the PRP mutants *albino seedling lethality 1* (*asl1*), *albino seedling lethality 2* (*asl2*), and *albino lethal 1*(*al1*) show albino and seedling lethality phenotypes (Gong et al. 2013; Lin et al. 2015; Zhao et al. 2016). *ASL1* encodes the plastid 30S ribosomal protein S20 (RPS20), *ASL2* encodes the chloroplast 50S ribosome protein L21, and *AL1* encodes the PRPL12 protein. *WLP1* encodes the plastid ribosomal protein L13 and *TCD11* encodes the small ribosomal subunit protein S6 (RPS6). The rice *white leaf and panicles 1* (*wlp1*) and *thermo-sensitive chlorophyll-deficient mutant 11* (*tcd11*) mutants display albino phenotypes at low temperatures, suggesting that both genes are required for normal chloroplast development, especially under low temperature conditions (Song et al. 2014; Wang et al. 2017a).

Although many PRPs have been studied in plants, few have been studied in rice. In the present study, we obtained a novel rice mutant, *white green leaf 2* (*wgl2*); this mutant exhibited an albino phenotype at the seedling stage and recovered green leaves at later stages. Map-based cloning revealed that *WGL2* encodes a ribosomal protein. Our results suggest that the PRP WGL2 plays a vital role in the early development of chloroplasts in rice.

## Results

### The *wgl2* mutants have an albino phenotype at the seedling stage

**Fig. 1:**
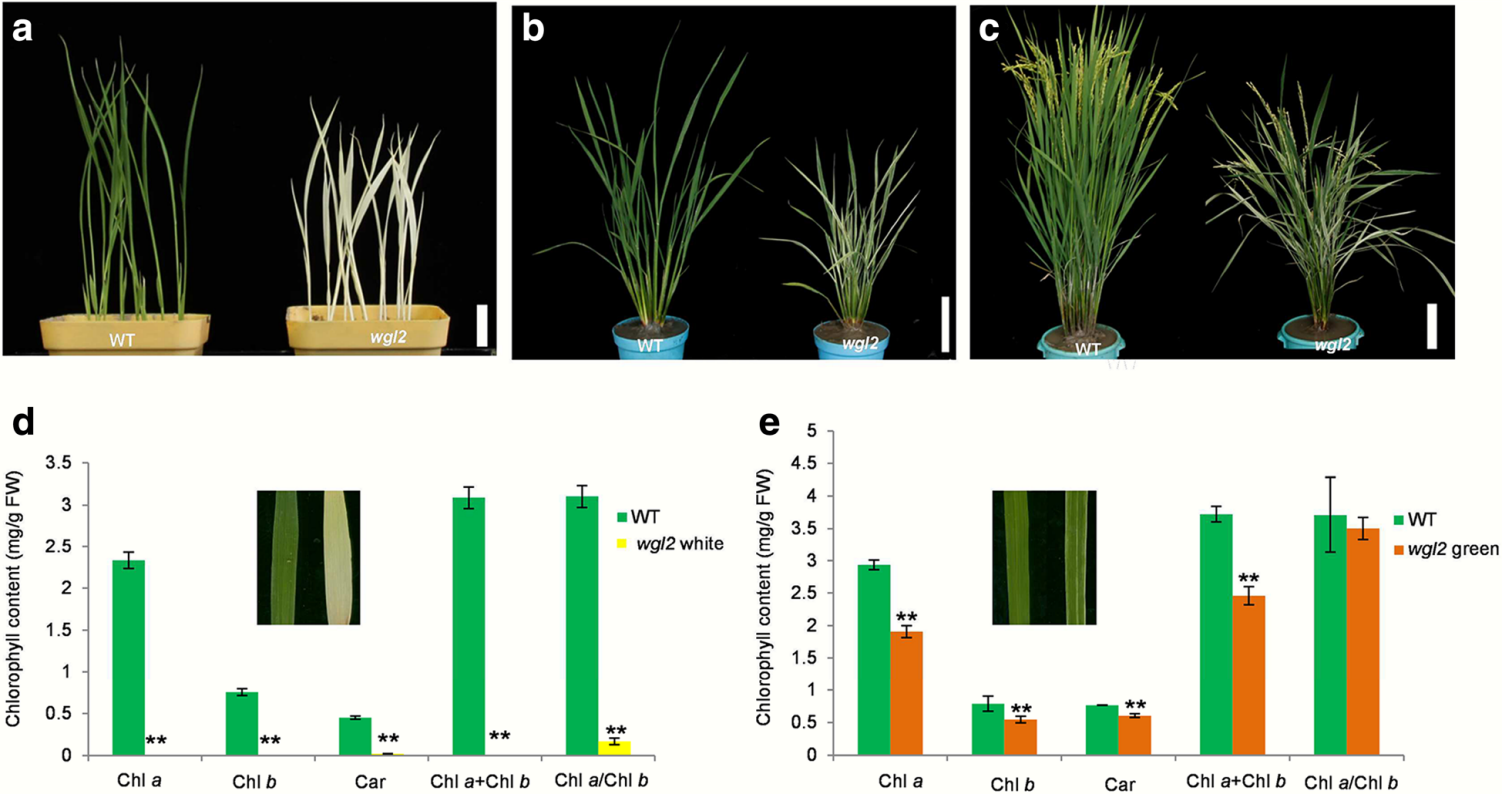
Phenotypes and pigment contents of wild type and *wgl2* plants. **a**-**c** Morphology of wild type (WT) and *wgl2* plants at the seedling (bar = 2 cm), tillering, and heading stages, respectively, in the paddy field (bar = 10 cm). **d** Pigment contents of WT and *wgl2* white seedlings. **e** Pigment contents of WT and *wgl2* green plants that older than the white ones. Chl *a*, chlorophyll *a*; Chl *b*, chlorophyll *b*; Car, carotenoids. Values represent the mean ± SD of 3 biological replicates. Student’s *t*-test was used to generate the *p* values; **p < 0.01

A novel rice albino mutant named *wgl2* was isolated from the *japonica* rice cultivar NPB that had been subjected to ethyl methane sulfonate mutagenesis. The *wgl2* mutant exhibited an albino phenotype from germination through the three-leaf stage, and then gradually turned from albino to green through the later stages of development (Fig. 1a, b, c). Consistent with the albino phenotype, *wgl2* mutants had lower Chl *a*, Chl *b*, and Car contents relative to the wild type (NPB). Moreover, the total chlorophyll content of *wgl2* and the ratio of Chl *a/b* were much lower than in the wild type (Fig. 1d). When *wgl2* exhibited green leaves at later stages of development, its Chl and Car contents recovered, but were still lower than wild type (Fig. 1e).

### *WGL2* affects chloroplast development

**Fig. 2:**
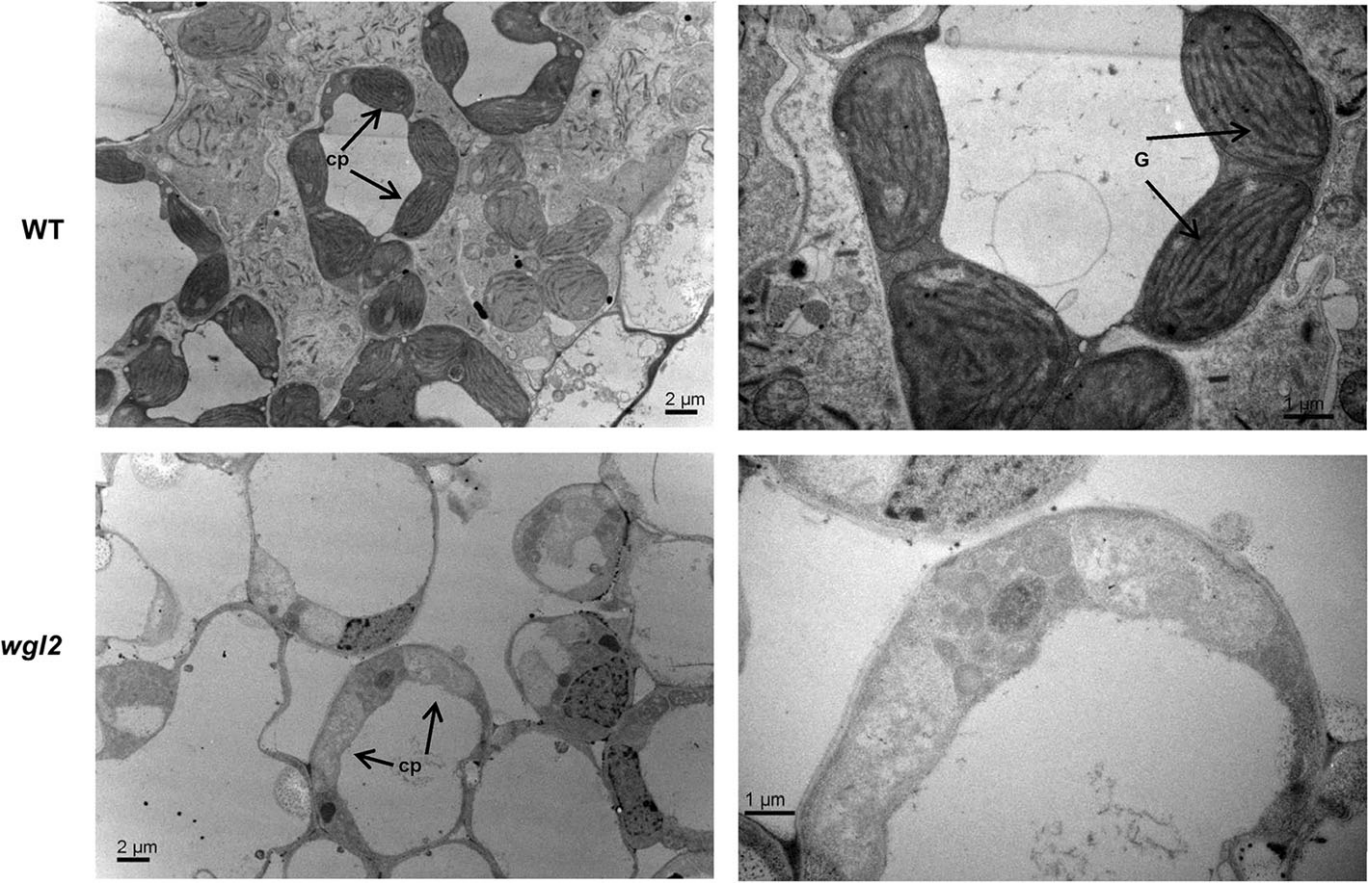
Microstructures of wild type (WT) and *wgl2* white seedlings chloroplasts viewed with transmission electron microscopy

Abnormal chloroplast development usually leads to lower total chlorophyll contents (Sakamoto et al. 2009). To examine whether chloroplast development was affected in *wgl2* albino seedlings, we analyzed the chloroplast ultrastructure of wild-type and *wgl2* seedlings at the three-leaf stage with transmission electron microscopy. We observed normal chloroplasts in wild-type plants, with dense and well-structured grana stacks. By contrast, the *wgl2* albino mutant had chloroplasts with disrupted architecture, as well as no stromal thylakoids or stacked grana thylakoids, instead displaying oval-shaped vesicles (Fig. 2). These results suggest that *wgl2* albino mutant is severely defective in chloroplast development.

### Map-based cloning and sequence analysis of *WGL2*

To explore the molecular mechanism responsible for the *wgl2* mutant phenotype, we generated an F_2_ population by crossing the *wgl2* mutant with the *indica* cultivar TN1 to generate a segregating population. All F_1_ plants displayed a normal green phenotype, and segregation occurred in the F_2_ plants that were selfed from the heterozygous F_1_ plants. Of the 528 F_2_ plants, 409 showed a normal green leaf phenotype and 119 showed the albino phenotype. The segregation ratio of wild-type to albino phenotypes among the F_2_ population was consistent with a 3:1 ratio (χ^2^ = 1.75 < χ^2^ 0.05 = 3.84). These results suggest that the *wgl2* mutant phenotype is determined by a single recessive nuclear gene.

**Fig. 3:**
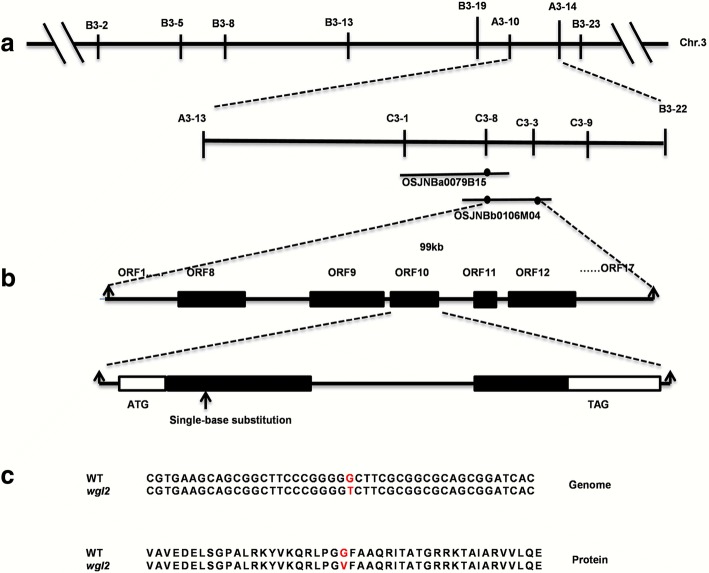
Map-based cloning and identification of the mutation site in *wgl2.* **a** The *WGL2* locus was initially mapped to Chromosome 3 (Chr. 3). **b** The *WGL2* locus was fine-mapped to a physical interval of 99 kb using the indicated markers. **c** Mutation site in *WGL2* at the genome and protein levels

Using 21 F_2_ mutant individuals, the *WGL2* locus was roughly mapped to the region between the markers A3–10 and A3–14 on the short arm of chromosome 3 (Fig. 3a). To fine map the *WGL2* locus, we designed additional sequence-tagged site markers. By using these and more than 1500 genotypes, the location of *WGL2* was narrowed down to a 99-kb region between markers C3–8 and C3–3, a region that includes 17 putative open reading frames (ORF: http://rice.plantbiology.msu.edu) (Fig. 3b, Additional file 1: Table S5). DNA sequence analysis of each putative ORF in the *wgl2* mutant revealed the presence of a single nucleotide substitution (G➔T) in the first exon of LOC_Os03g55930, which resulted in a Gly-to-Val substitution at the 92nd residue (G92 V, Fig. 3c). The gene is 672 bp long, includes two exons and one intron, and encodes a 224 amino acid polypeptide (Fig. 3b). We used Swiss Model (http://swissmodel.expasy.org/) to predict three-dimensional structures for both wild-type and mutant forms of the WGL2 protein and did not observe any obvious structural differences (Biasini et al. 2014).

### Complementation and targeted deletion tests

**Fig. 4:**
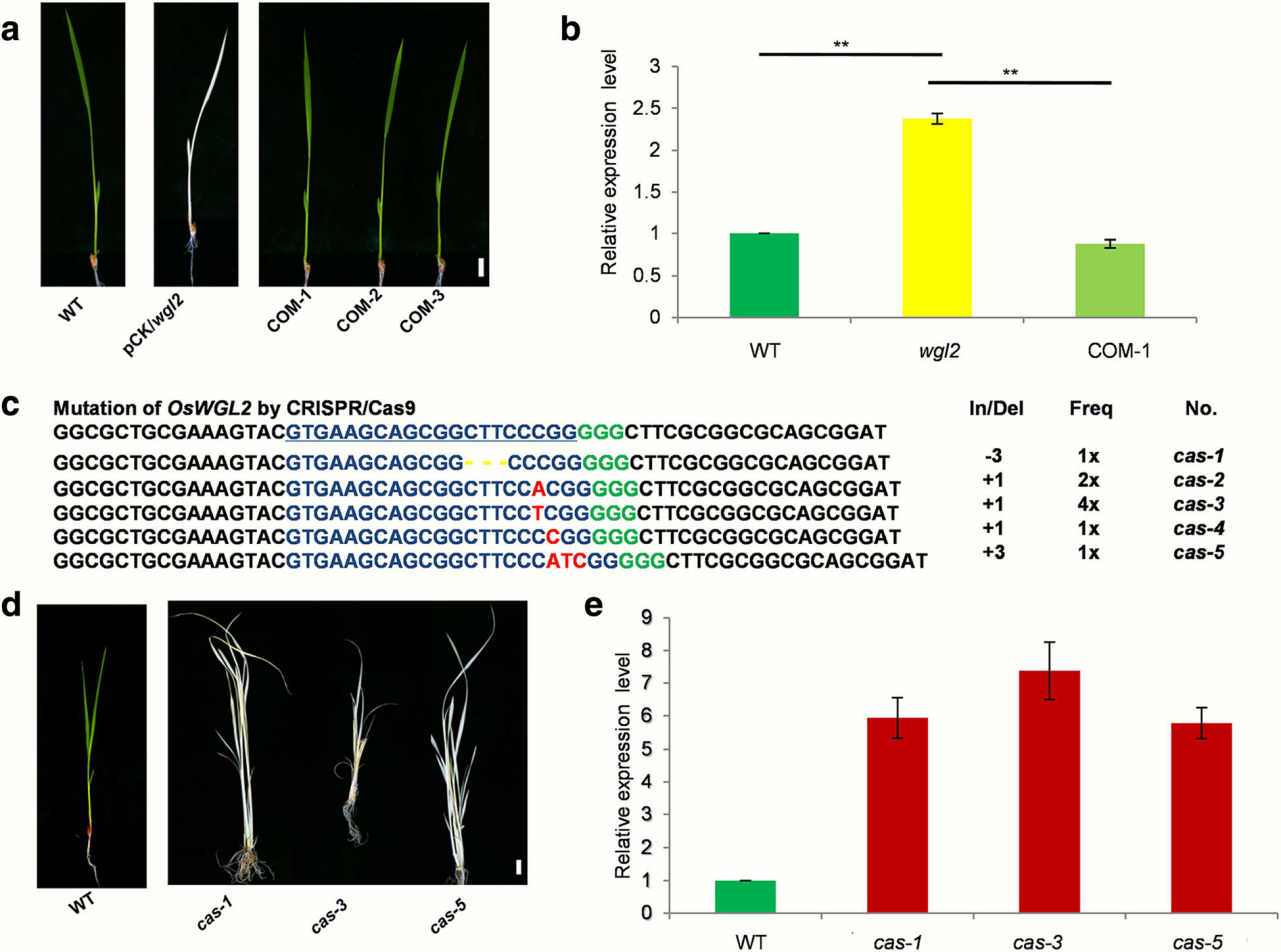
Complementation and targeted deletion experiments. **a** From left to right: wild type (WT), *wgl2* with pCAMBIA1300 (pCK/*wgl2*), and seedlings from three independent *WGL2* complementation lines (COM-1, 2, and 3) (bar = 1 cm). **b** Relative *WGL2* transcript levels in WT, *wgl2*, and complementation transgenic plants. **c** Sequence confirmation of the *WGL2* deletion in WT plants harboring Cas9/sgRNA constructs. The 20-nt target sequence of the Cas9/sgRNA complex is underlined in blue, and the PAM site is indicated in green. For the Cas9/sgRNA-mutated DNA sequences, inserted nucleotides are shown in red, and deleted nucleotides are depicted as yellow dots. **d** Phenotypes of mutant seedlings harboring the *WLG2* Cas9/sgRNA constructs (bar = 1 cm). **e** Relative transcript levels of *WGL2* in WT and three independent mutant transgenic plants. The *WGL2* transcript levels in WT were set to 1. Values represent the mean ± SD of 3 biological replicates

To confirm that the albino phenotype was attributable to the detected mutation in *WGL2*, we constructed a complementation vector with a wild-type genomic fragment containing the entire coding region of *WGL2*, along with 2122 bp of upstream sequence and 281 bp of downstream sequence. The fragment was inserted into the binary vector pCAMBIA1300 to make pCAMBIA1300-*WGL2* (Additional file 1: Table S3), which was introduced into the *wgl2* mutant by *Agrobacterium*-mediated transformation. As expected, 15 independent transgenic T_0_ lines (pCAMBIA1300-*WGL2*/*wgl2*) showed a normal phenotype, whereas the *wgl2* plants that were transformed with the empty vector showed an albino phenotype that was similar to that of the *wgl2* mutant (Fig. 4a). Consistent with this, the T_1_ plants derived from the T_0_ plants (pCAMBIA1300-*WGL2*/*wgl2*) displayed either green or albino phenotypes, and all of the green seedlings were found to contain the transgene.

Additionally, we used CRISPR/Cas9 to generate mutant alleles of *WGL2* in a wild-type background and obtained 9 independent T_0_ transgenic lines that all carried homozygous mutations. Although each line with a homozygous *OsWGL2* phenocopied the *wgl2* mutant, it exhibited a seedling lethal phenotype. (Fig. 4c, d). Quantitative RT-PCR analysis demonstrated that *WGL2* expression in pCAMBIA1300-WGL2/*wgl2* complemented transgenic plants was similar to that in the wild type, whereas *WGL2* transcript levels were increased in the *wgl2* mutant and in all three independent *wgl2cas* lines compared to the wild type (Fig. 4b, e). These results support the idea that *WGL2* is LOC_Os03g55930 and its mutation is responsible for the *wgl2* mutant phenotype.

### Subcellular localization of WGL2

**Fig. 5:**
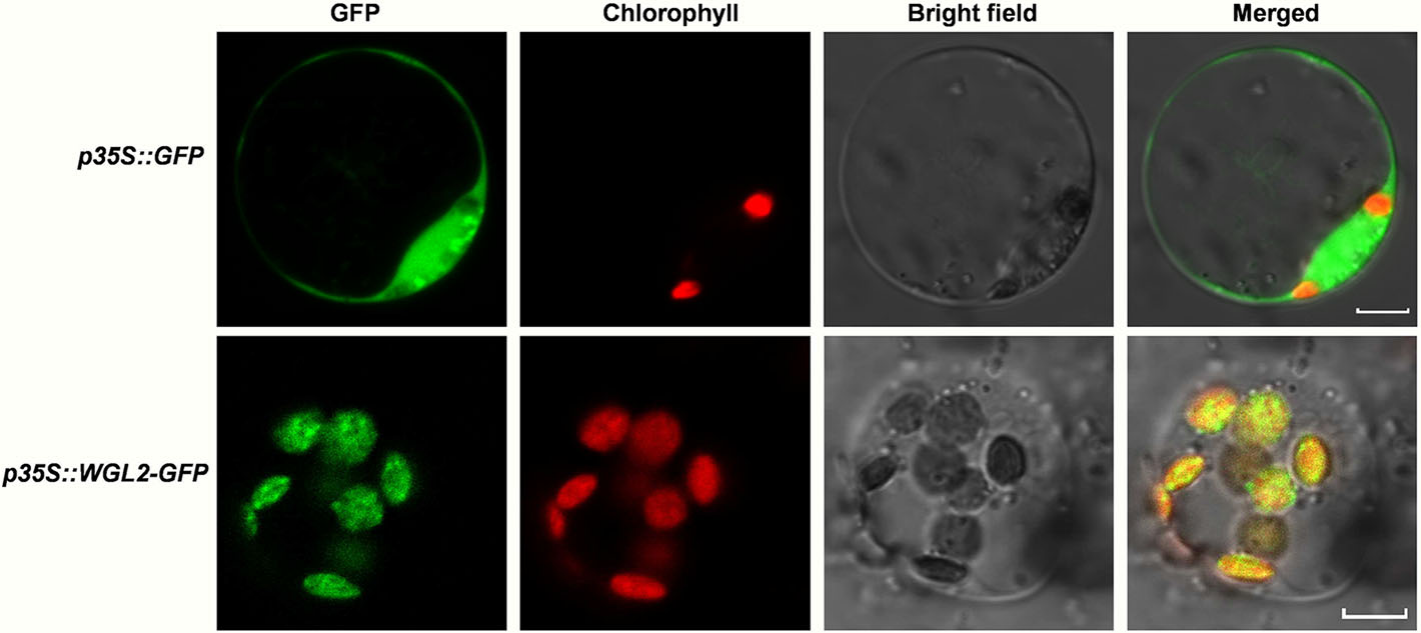
Subcellular localization of the WGL2-GFP protein in rice protoplasts. GFP signals show that the WGL2-GFP fusion protein produced from the *p35S::WGL2-GFP* construct localized to the chloroplast and the control GFP localized to the cytoplasm and nucleus. Green fluorescence shows GFP, red fluorescence shows chloroplast autofluorescence, and yellow fluorescence shows the merged fluorescence. (bar = 5 μm)

To explore the subcellular localization of WGL2, the full-length *WGL2* coding sequence lacking its stop codon was used to construct the pWGL2-GFP vector (Additional file 1: Table S3). The pWGL2-GFP plasmid was transformed into rice protoplasts, and an empty GFP vector was used as a negative control. The green fluorescent signal of pWGL2-GFP transformants overlapped with the chlorophyll autofluorescence signal from chloroplasts, whereas the empty GFP vector was observed in the cytoplasm and the nucleus (Fig. 5). These results affirm that WGL2 localizes to the chloroplast.

### Phylogenetic analysis of WGL2

**Fig. 6:**
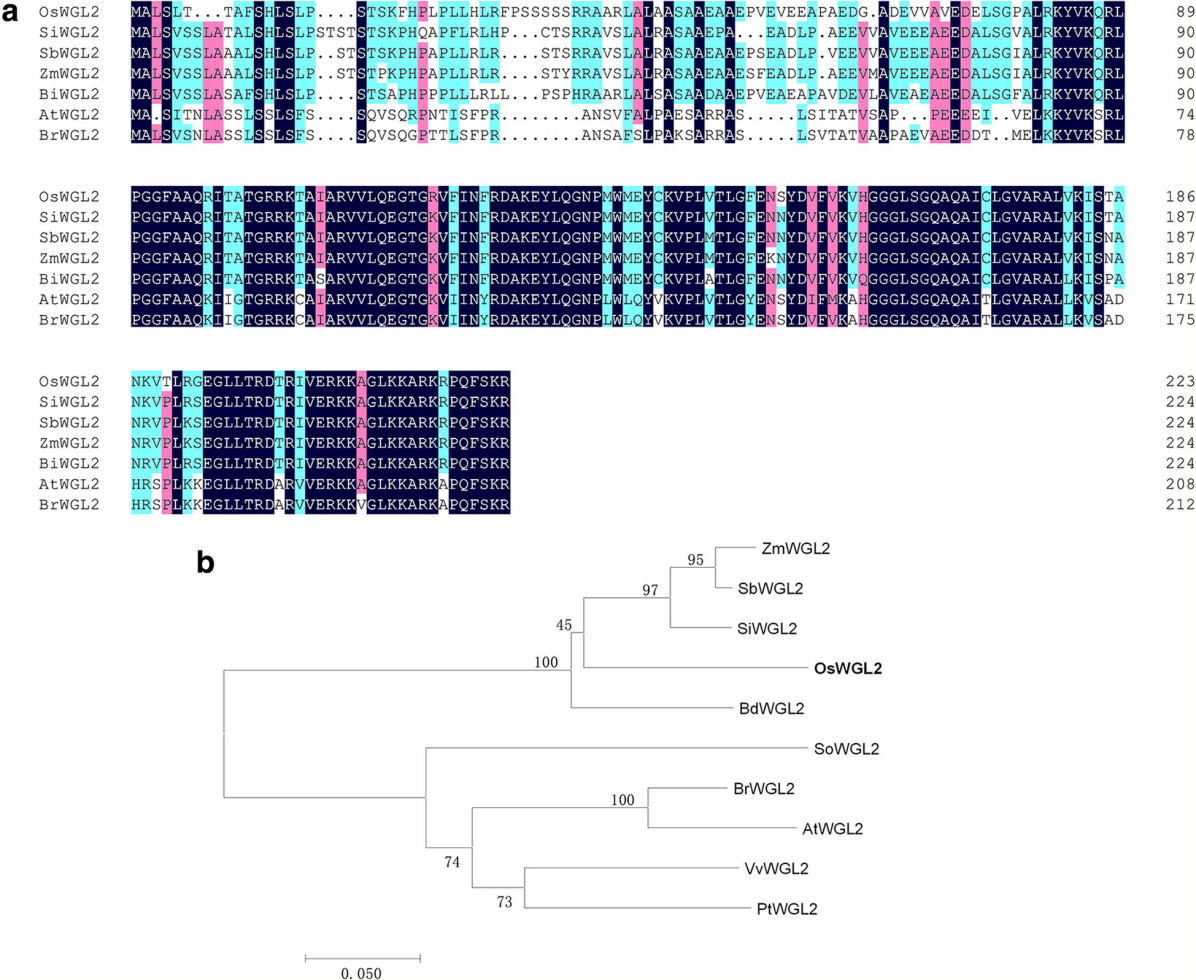
Phylogenic analysis of WGL2. **a** Amino acid sequence alignment of the 7 types of WGL2 homologs. Amino acids that were fully or partially conserved are shaded blue and green, respectively. **b** Phylogenic tree of OsWGL2 and its homologs. Protein sequences are *Setaria italica* (SiWGL2*,* XP_004981623.1), *Sorghum bicolor* (SbWGL2, XP_002463825.1), *Zea mays* (ZmWGL2, NP_001105916.2), *Brachypodium distachyon* (BdWGL2, XP_003558826.1), *Brassica rapa* (BrWGL2, XP_009104732.1), *Arabidopsis thaliana* (AtWGL2, BAD44402.1), *Spinacia oleracea* (SoWGL2, XP_021845391.1), *Vitis vinifera* (VvWGL2, XP_002283650.1), *Populus trichocarpa* (PtWGL2, XP_006375046.1). The tree was constructed using MEGA version 7.0. Scale represents percentage substitutions per site. Statistical support for the nodes is indicated

The C-terminal region of chloroplast proteins is often crucial for protein stability, binding with other proteins, photophosphorylation, and trafficking to the chloroplast envelope (Bals et al. 2010; Dünschede et al. 2011; Lung and Chuong 2012; Urbischek et al. 2015). BLAST-P analysis of the NCBI database showed that the WGL2 C-terminal belongs to the ribosomal S9 super family, and is highly conserved in higher plants including *Setaria italica*, *Zea mays*, *Brachypodium distachyon*, *Brassica rapa*, and *Arabidopsis thaliana* (Fig. 6a). Among these species, WGL2 from rice exhibited the highest similarity to the orthologs in *Sorghum bicolor* (81%), *Setaria italic* (80%), and *Zea mays* (79%). To investigate the evolutionary relationship among WGL2 homologs, a phylogenic analysis was performed (Fig. 6b). The results show that WGL2 homologues can be clearly divided into two groups: monocots and dicots, and that OsWGL2 is closely related to the grass family containing *maize* and *sorghum*. These results demonstrate that the WGL2 protein is a ribosomal S9 protein that is highly conserved in plants.

### *WGL2* is predominantly expressed in the leaf

**Fig. 7:**
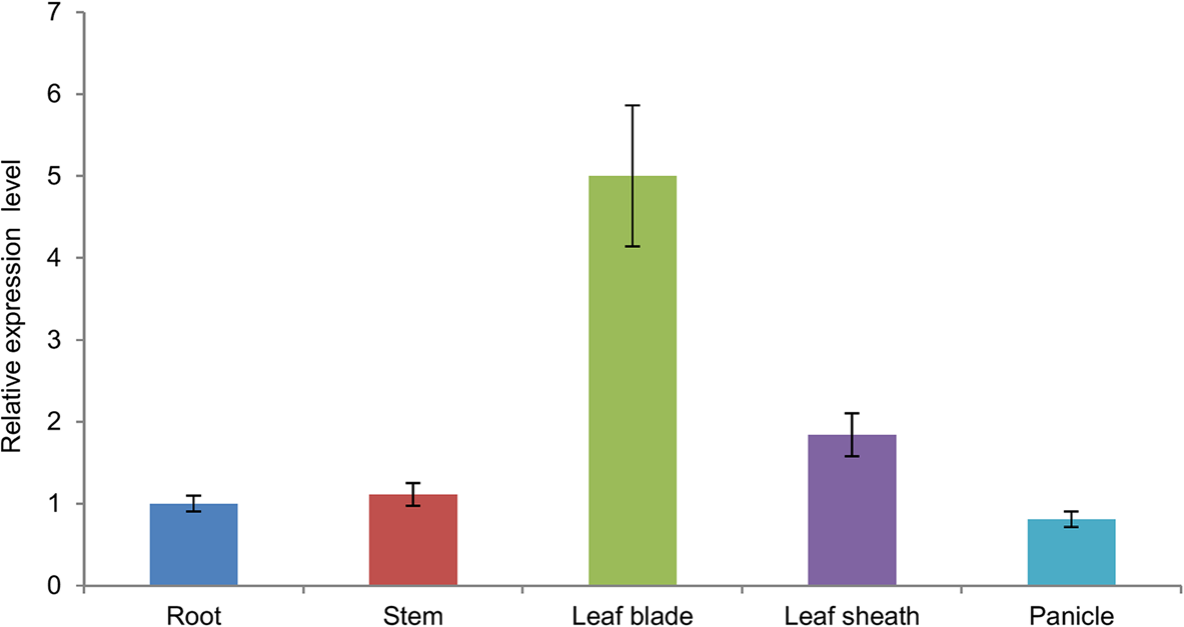
Relative *WGL2* transcript levels in various organs of WT plants. The *WGL2* transcript levels in the roots were set to 1. Values represent the mean ± SD of 3 biological replicates

To examine the *WGL2* expression pattern in wild-type plants, total RNA was extracted from various organs including young roots, uppermost internodes, flag leaves, leaf sheaths, and young panicles. We detected a dramatic increase in *WGL2* expression in flag leaves, and a slight increase in the leaf sheath compared to that in the other organs (Fig. 7). This further supports a role for *WGL2* in chloroplast development in rice leaves.

### The expression of genes related to chloroplast development, photosynthesis, and chloroplast RNAs is affected in the *wgl2* mutant

**Fig. 8:**
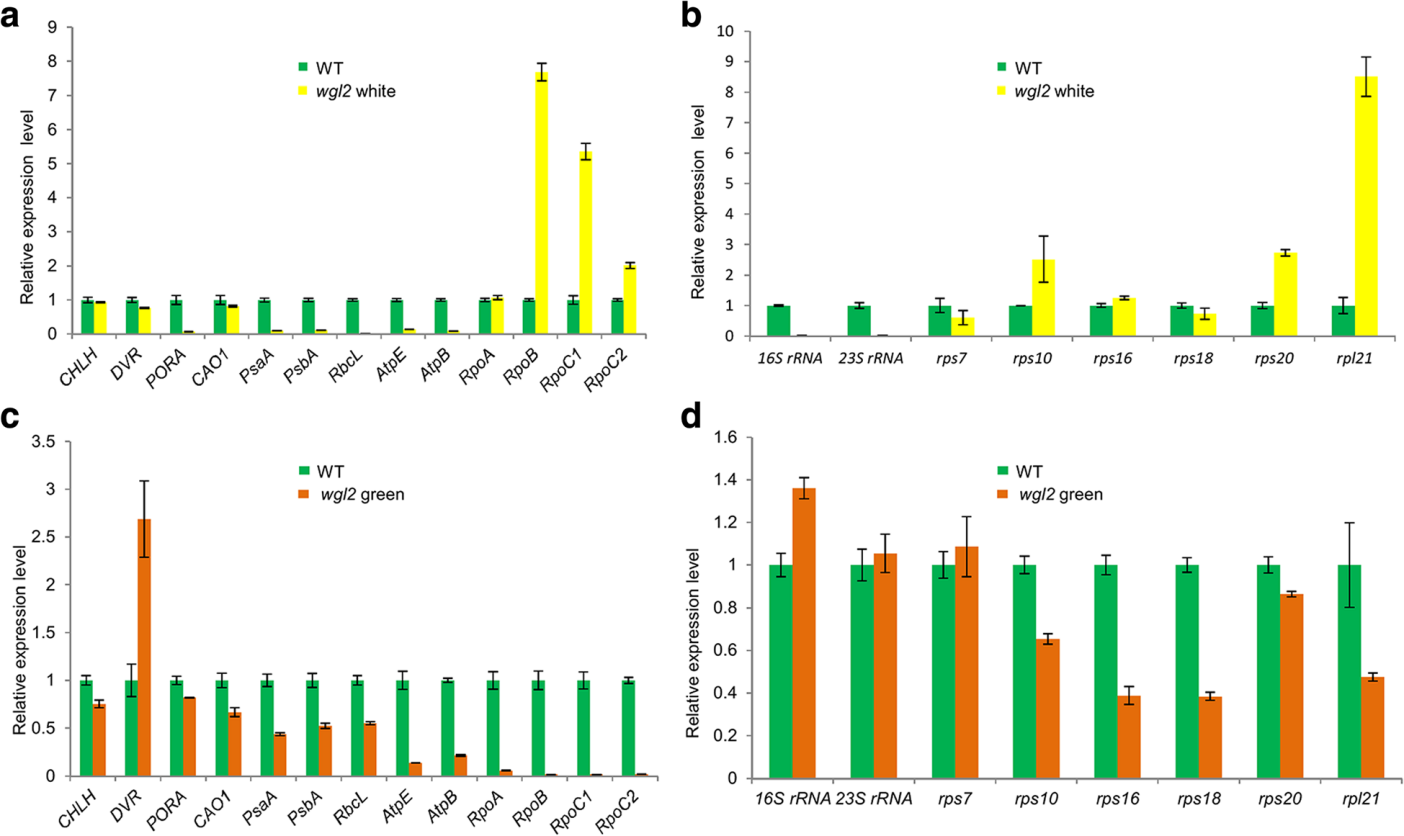
Expression of genes associated with Chl biosynthesis, photosynthesis, chloroplast development, and ribosome development in the *wgl2* mutant and wild type. **a**, **c** Expression of genes associated with Chl biosynthesis, photosynthesis, chloroplast development in WT and *wgl2* white seedlings (**a**), and in WT and *wgl2* green plants (**c**). **b**, **d** Expression of genes associated with ribosome development in WT and *wgl2* white seedlings (**b**), and in WT and *wgl2* green plants (**d**). The relative expression level of each gene was normalized using *UBQ5* as an internal control. The expression level of each gene in WT was set to 1.0 and other samples were calculated accordingly. Values represent the mean ± SD of 3 biological replicates

Given that WGL2 is a chloroplast nucleoid-localized ribosomal protein, we considered the possibility that it might affect the expression of genes related to chloroplast development. Therefore, we measured transcript levels of 13 representative genes associated with Chl biosynthesis, photosynthesis, and chloroplast development with qRT-PCR (Fig. 8a, c). The transcript levels of the Chl biosynthesis genes encoding Mg chelatase H subunit (*CHLH*), chlorophyll a oxygenase1 (*CAO1*), protochlorophyllide A (*PORA*), and divinyl reductase (*DVR*) decreased in the *wgl2* albino leaves compared to the wild type (Fig. 8a). However, the transcript level of *DVR* in *wgl2* green leaves that were showed at later stages of development was 2.7 times higher than in wild-type leaves (Fig. 8c).

Chloroplast biogenesis and physiological changes are tightly regulated by the coordination of NEP and PEP. We observed that the expression of PEP-dependent photosynthesis genes including *psaA*, and *psbA*, which encode core components of Photosystem I, and *rbcL*, which encodes the large subunit of Rubisco, were lower in *wgl2* albino leaves than in the wild-type leaves (Fig. 8a). The expression of NEP-dependent genes, including the RNA polymerase subunit genes *RpoB*, *RpoC1*, and *RpoC2* was higher in *wgl2* albino leaves compared with the wild type. Moreover, the *AtpB* and *AtpE* NEP/PEP-dependent genes were strongly inhibited in *wgl2* albino leaves compared with the wild type. The expression levels of these genes in *wgl2* green leaves were different from the levels in *wgl2* albino leaves and in wild-type leaves. For example, the transcript levels of the RNA polymerase subunit genes *RpoB*, *RpoC1*, and *RpoC2* were obviously lower in *wgl2* green leaves compared with wild-type leaves (Fig. 8c).

In addition, we examined the expression of genes related to ribosome development (Fig. 8b, d). We found that 16S rRNA, 23S rRNA, *rps7*, and *rps18* were severely suppressed in the *wgl2* albino mutant compared with the wild type, especially the 16S rRNA and 23S rRNA that were almost undetectable. Nevertheless, *rps10*, *rps16*, *rps20*, and *rpl21* were up-regulated in the *wgl2* albino mutant, of which, the increase in *rpl21* expression was the most dramatic. When the expression levels of these genes were detected in *wgl2* green leaves and wild-type, 16S rRNA levels were higher in *wgl2* green leaves than in wild-type, and 23S rRNA and *rps7* were similar between *wgl2* green leaves and wild type; however, the remaining genes were down-regulated in *wgl2* green leaves compared with the wild-type. These data show that the mutation in *WLG2* results in abnormal expression of genes related to chloroplast development, photosynthesis, and chloroplast RNAs, which may be responsible for the albino phenotype observed in *wlg2* albino seedlings.

## Discussion

When chloroplast development and chlorophyll biosynthesis are disrupted, plants usually exhibit a variety of leaf color phenotypes (Jung et al. 2003). Plastid ribosomal proteins are crucial in ribosome biogenesis, plastid protein biosynthesis, and early chloroplast development (Lin et al. 2015). Many plastid ribosomal protein mutants have been identified in higher plants, and certain plastid ribosomal proteins have been shown to have roles in diverse biological processes in Arabidopsis (Romani et al. 2012). However, few studies have investigated the functions of PRPs in rice. The first rice PRP mutant reported was the *asl1* mutant, which shows an albino lethal phenotype at the seedling stage (Gong et al. 2013). The nuclear gene *ASL1* encodes PRPS20 and the *asl1* mutation results in abnormal expression of genes related to chlorophyll biosynthesis, photosynthesis, chloroplast development, and plastid ribosome assembly. The rice PRP mutants *asl2* and *albino lethal 1* (*al1*) also show similar albino and seedling lethal phenotypes, as well as a reduction in expression of the 23S rRNA involved in plastid ribosome assembly. *ASL2* and *AL1* encode PRRL21 and PRPL12, respectively, both of which participate in ribosome assembly (Lin et al. 2015; Zhao et al. 2016). Additionally, the rice PRP mutant *white leaf and panicles 1* (*wlp1*) displays an albino phenotype at low temperatures, which is similar to the *thermo-sensitive chlorophyll-deficient mutant 11* (*tcd11*) mutant. *WLP1* encodes PRPL13, and *TCD11* encodes PRPS6. Both *WLP1* and *TCD11* are necessary for normal chloroplast development, especially at low temperatures (Song et al. 2014; Wang et al. 2017a).

In this study, a seedling albino rice *wgl2* mutant was identified and consistent with its albino phenotype, showed altered pigment content and disordered chloroplast development (Figs. 1a, d and 2). The *wgl2* mutant had a single nucleotide substitution (G➔T) in the first exon of LOC_Os03g55930, which resulted in a Gly-to-Val substitution at 92nd residue (Fig. 3c). According to the RGAP database (http://rice.plantbiology.msu.edu/index.shtml), *WGL2* encodes a ribosomal protein. We found that the WGL2 protein localizes to the chloroplast (Fig. 5). Therefore, we supposed that WGL2 is a type of PRP. Furthermore, a BLASTP analysis in the NCBI database showed that the C-terminal of WGL2 belongs to the ribosomal S9 super family and it is highly conserved among chloroplast 30S ribosomal S9 proteins of higher plants (Fig. 6a). According to the NCBI database, *WGL2* is the only gene that encodes a ribosomal S9 protein in rice.

The process of plastid formation from a proplastid to a photosynthetically active chloroplast is controlled by NEP and PEP (Hedtke et al. 1997; Shiina et al. 2005). The coordination of NEP and PEP expression plays an important role in chloroplast differentiation. The transcript levels of 13 representative genes associated with Chl biosynthesis, photosynthesis, and chloroplast development were assessed by qRT-PCR (Fig. 8a, c). We found that the expression of PEP-dependent photosynthesis genes, such as *psaA*, *psbA*, and *rbcL* were severely reduced in the *wgl2* mutant, which could possibly be related to the deficiency in chloroplast ribosomes. However, the transcript levels of NEP-dependent genes, such as the RNA polymerase subunits *RpoB*, *RpoC1*, and *RpoC2* increased in *wgl2* albino mutant compared with the wild-type (Fig. 8a), decreased in *wgl2* green seedlings compared with the wild-type (Fig. 8c). This increase in expression of NEP genes may be caused through feedback mechanisms to overcome the reduced translation of PEP genes in the chloroplast because NEP preferentially transcribes plastid house-keeping genes, including PEP core subunits and genes associated with basic plastid functions. In addition, the decrease in transcripts of nuclear genes involved in Chl biosynthesis (*CHLH*, *CAO1* and *PORA*) is probably due to the disruption of chloroplast development at the chloroplast build-up step. Consequently, the *wgl2* albino or green plants have lower Chl *a*, Chl *b*, and Car contents than the wild type (Fig. 1d, e).

A BLAST-P analysis using the NCBI database shows that WGL2 has high sequence identity with chloroplast 30S ribosomal protein S9 from multiple plant species. Our observations show that the expression of the chloroplast-associated 16S and 23S rRNAs were dramatically reduced in the *wgl2* albino mutant compared to the wild type, and that other genes related to ribosome development also displayed abnormal expression (Fig. 8b). However, most of these genes expression levels in *wgl2* green and *wgl2* albino were opposite, especially 16 rRNA, *rps10*, *rps20*, and *rpl21* (Fig. 8b, d). The transcript abundance of *WGL2* was higher than the wild type in both the *wgl2* point mutant and the CRISPR/Cas9 transgenic *wgl2* mutant lines, which may result from feedback effects (Fig. 4b, e). These results are similar to those observed for the rice *wlp1* and *al1* mutants (Song et al. 2014; Zhao et al. 2016), and show that mutation of the *WGL2* gene hinders Chl biosynthesis, photosynthesis, chloroplast development, and ribosome development in chloroplasts, thereby leading to the observed phenotype in the *wgl2* mutant.

RPS9 is widely conserved in yeast, bacteria, protists, mammals, humans, and plants (Wen et al. 2017). RPS9 plays crucial roles in many biological processes such as the early stages of ribosome biogenesis, regulation of mRNA translation, and normal cell growth and proliferation (Ferreiracerca et al. 2007; Lindström and Nistér 2010; Lindström and Zhang 2008; Pnueli and Arava 2007). RPS9 in rice has high sequence identity with maize PRRS9, which is disrupted by the *lethal embryo 1* (*lem1*) mutant; however, the *lem1* mutant displays an early embryo lethality phenotype instead of the albino phenotype observed in the *wgl2* mutant in rice (Ma and Dooner 2004). In contrast, the null *rps9* mutant in bacteria is not lethal, but has a cold-sensitive phenotype. Since single-base substitution is not lethal in the rice *wgl2* mutant, the mutant WGL2 protein may be partially functional. Interestingly, we used the CRISPR/Cas9 system to obtain nine independent homozygous T_0_ transgenic lines, which show albino and seedling-lethal phenotypes, indicating that the *OsWGL2* loss-of-function hinders chloroplast development and fails to recover the albino seedling phenotype. These phenotypic differences may be due to different functions of RPS9 in different species and different mutations of RPS9 in the same species.

Many studies have shown that leaf color mutants can transition from abnormal phenotypes to normal phenotypes, and that this transition is primarily affected by environmental conditions, especially light and temperature. The rice *temperature-sensitive virescent* (*tsv*) mutant displays a normal green phenotype at high temperatures, whereas the rice *heat-stress sensitive albino 1* (*hsa1*) mutant shows normal phenotypes at low temperatures (Qiu et al. 2017; Sun et al. 2017). Light is an important factor in the differentiation of non-photosynthetic proplastids into fully functional, photosynthetic chloroplasts, and affects the expression of genes associated with chloroplast development (Gong et al. 2013; Su et al. 2012; Zhao et al. 1999). The transcript levels of *ASL1* and *ASL2* were higher when exposed to light than in the dark (Gong et al. 2013; Zhao et al. 2016). In this study, the rice *wgl2* mutant exhibited the albino phenotype from germination through the three-leaf stage, and then gradually turned from albino to green during the later stages of development (Fig. 1a, b, c), which may be also affected by environmental conditions. However, further experiments will need to be done to investigate the impact of light and temperature on the *wgl2* mutant phenotype to elucidate the role of these environmental factors.

## Conclusions

Rice *WGL2* encodes a conserved ribosomal protein, which localizes to the chloroplast. *WGL2* is essential for early chloroplast development in rice. These results will facilitate efforts to further uncover the molecular mechanism of chloroplast development.

## Methods

### Plant materials growth conditions

The *wgl2* mutant was obtained by mutagenizing the *Oryza sativa japonica* cultivar Nipponbare (NPB) with ethyl methane sulfonate (Qian et al. 2016). The F_2_ segregating population was generated by crossing the *wgl2* mutant with the *indica* cultivar Taizhong Native 1 (TN1) and was used for fine mapping *wgl2*. All plants in this study were grown under natural conditions in the paddy field at the China National Rice Research Institute located in Fuyang, Zhejiang province (119°6′E, 30°0′N).

### Measurement of pigment content

Pigments were extracted from fresh leaf samples (0.03 g) from wild-type and *wgl2* plants that were cut into small segments and incubated with 3 ml of 80% acetone in the dark for 48 h. The supernatant was measured with an ultraviolet spectrophotometer (DU800, BECKMAN, Fullerton, USA) at 470, 645, and 663 nm. Total chlorophyll (Chl), Chl *a*, Chl *b*, and carotenoid (Car) contents were measured according to the methods by Arnon (1949) and Wellburn (1994) (Arnon 1949; Wellburn 1994). Three biological replicates were analyzed for each condition.

### Transmission electron microscopy (TEM)

For TEM analysis, transverse sections of leaf samples were taken from the *wgl2 white* and NPB seedlings grown in the paddy field at the three-leaf stage. Leaf samples were fixed in 2.5% glutaraldehyde and then in 1% OsO_4_. Samples were prepared as described previously (Gothandam et al. 2005), and were observed using a Hitachi H-7650 electron microscope (Tokyo, Japan).

### Map-based cloning and vector construction

To map the *WGL2* gene, more than 1500 mutant individuals were selected from the F_2_ population. Genomic DNA (gDNA) was extracted from F_2_ plants with the CTAB method (Jr and Via 1993). Polymorphic markers between NPB and TN1 were identified using simple sequence repeat (SSR) and sequence-tagged site (STS) markers, and were used to map the *wgl2* locus. PCR products were separated by agarose gel (4–5%) electrophoresis. The gDNA fragments containing candidate genes were amplified from mutant and WT and sequenced by the Hangzhou Tsingke Biological Engineering Technology and ServiceCo. Ltd. (Tsingke, Hangzhou, China).

For mutant complementation, the gDNA sequence containing the entire coding region of *WGL2*, 2122-bp upstream and 281-bp of downstream was amplified and sequenced with the 930COMF/R primers (Additional file 1: Table S3). The resulting fragment was inserted into the binary vector pCAMBIA1300. The pCAMBIA1300-WGL2 vector and control vector (pCAMBIA1300, CK) were introduced into the *wgl2* mutant by *Agrobacterium*-mediated transformation. For making *wgl2* mutants, the target sequence (GTGAAGCAGCGGCTTCCCGG) was chosen using CRISPRdirect (http://crispr.dbcls.jp) and then confirming its specificity (Naito et al. 2015). For construction of the targeted deletion vector, the first round of PCR was performed in two separate reactions with U3tF/tR1tR- WGL2 and gR2tF-WGL2/gRNA2R2, and the second round of PCR was performed with U3tF/ gRNA2R2 (Additional file 1: Table S3).The targeted deletion phRubi2-Cas9 vector was constructed for the CRISPR/Cas9 system (Wang et al. 2017b) and was introduced into NPB by *Agrobacterium*-mediated transformation.

### Phylogenetic analysis

The predicted full-length WGL2 protein sequence, with 224 amino acids, was obtained from Gramene (http://www.gramene.org/). The sequences used in the phylogenetic analysis were obtained by a BLASTP search using the WGL2 protein sequence as the query at the National Center for Biotechnology Information (NCBI, http://www.ncbi.nlm.nih.gov/). The full-length amino acid sequences were aligned using the DNAMAN program. A neighbor-joining tree was constructed using MEGA version 7.0 software with the bootstrap method and 1000 replicates. The evolutionary distances were computed using the Poisson correction method and reflect the number of amino acid substitutions. All positions containing gaps and missing data were eliminated.

### Subcellular localization

To investigate the subcellular localization of WGL2, we amplified the full-length *WGL2* coding sequence without the termination codon with primers WGL2-GFPF/R (Additional file 1: Table S3). The resulting fragment was introduced into the GFP vector pCA1301-35S-S65 T-GFP (Ren et al. 2017). The WGL2-GFP vector was transformed into rice protoplasts (Yu et al. 2014), and the transformed protoplasts were observed with a Zeiss LSM700 laser scanning confocal microscope (Car Zeiss, Inc., Thornwood, NY, USA).

### RNA extraction and qRT-PCR analysis

Total RNA was extracted and purified from different tissues using the AxyPrep total RNA Miniprep Kit (Axygen) according to the manufacturer’s instructions. The cDNA was reverse transcribed using the ReverTraAce quantitative PCR RT Master Mix Kit with gDNA remover (Toyobo) according to the manufacturer’s instructions. Quantitative reverse transcription PCR (qRT-PCR) was performed with a CFX96 Touch Real-time PCR Detection System using the 2× SsoFast EvaGreen SuperMix (Bio-Rad). The rice *UBQ5* gene was used as an internal control. All primers for qRT-PCR are listed in Additional file 1: Table S2. The data were expressed as the mean ± SD of three biological replicates. A Student’s *t*-test was used for statistical analysis.

## Additional file

Additional file 1: **Table S1.** Markers used for fine mapping and sequencing of the WGL2 locus. **Table S2.** RT-PCR primers. **Table S3.** Vector construction primers. **Table S4.** Effect of mutations in cas on WGL2. **Table S5.** Predicted genes in the mapped region. (DOCX 31 kb)
