# Supplementary material for: The rice white green leaf 2 gene causes defects in chloroplast development and affects the plastid ribosomal protein S9
Source: Rice (N Y). 2018 Jul 11;11:39. doi: 10.1186/s12284-018-0233-2 (PMC6041223; doi:10.1186/s12284-018-0233-2)
Supplement: Supplementary file 1 — Table S1. Markers used for fine mapping and sequencing of the WGL2 locus. Table S2. RT-PCR primers. Table S3. Vector construction primers. Table S4. Effect of mutations in cas on WGL2. Table S5. Predicted genes in the mapped region. (DOCX 31 kb) [file 12284_2018_233_MOESM1_ESM.docx]

Additional file 1: Table S1: Markers used for fine mapping and sequencing of the *WGL2* locus

| Marker | Sense primer (5′–3′) | Antisense primer (5′–3′) |
| --- | --- | --- |
| A3-10 | TAAAAACCTCACCTCGCTGG | TTCGTTCACTCAGTGGCTTG |
| A3-13 | AGGAGCAAGAAAAGTTCCCC | GCCAATGTGTGACGCAATAG |
| A3-14 | GTCCCGAACCCTAGCCCGAGGG | AGAGGCCCTCCACATGGCGACC |
| B3-2 | TGTTGTTTTGGAGATTTGAAGG | GAGGCGAAAAGTCACGTAAGC |
| B3-5 | TGGCCGTTGAGGCGATTAG | CAGAGTCTTACTGCTAACCCCGT |
| B3-8 | CAGCTAGGATGTTGAAGGATCG | GCCAGCTTTGACTGCACTGC |
| B3-19 | ATCAGCAAGAAAGCTCTGCTCC | AGGAAATTCGCCCTAGTAGATAG |
| B3-22 | GCAAGACGAGGCAAGGCTG | AGTTGTACTCTGGTTGCTTCCGC |
| C3-1 | TGGGCTTGTATTATATTGGACG | GGTCCAGTCCAGGGTAGTAAAG |
| C3-3 | GTTAAAATTTCCCCCCGTA | TCTCCACCGTCCGATCTC |
| C3-8 | TCAGAGAACAGCAAATCGGT | CCAGGGAGGAGAAGAATCAT |
| C3-9 | GTCAAGCTGACTCCCAAGC | CAAGAAAGACACTCTCCCCAG |

Additional file 1: Table S2: RT-PCR primers.

| Gene | Sense primer (5′–3′) | Antisense primer (5′–3′) |
| --- | --- | --- |
| *WGL2* | CGCTGCGAAAGTACGTGAAG | AATACTCCTTGGCATCGCGG |
| *CHLH* | TGACTCAGACCCGACAAAGC | TCCCCTCGTACCACTTAGGG |
| *DVR* | CAGGTCGAGACCGTCAAGAAC | ATGACCTGGATCGGCACCTTG |
| *PROA* | TGTACTGGAGCTGGAACAACAACT | TCAATAGCACATCACTCTCACTCACT |
| *CAO1* | GACACCTTCATCTGGGCTTCAA | CGAGAGACATCCGGTAGAGC |
| *PsaA* | GTTTTCGCGGAGGGCTAGAT | TGACCTGCGATCAGGAAAAGA |
| *PsbA* | ACTAGCACCGAAAACCGTCTTT | CAGCGATGAAGGCGATAATAAA |
| *RbcL* | CTCGCGGTATCTTTTTCACTCA | TCGGTCAGAGCTGGCATATG |
| *AtpB* | TGAGAGGAATGGAAGTGATTGACA | TCAACAGGCTCCCCAAGAAC |
| *AtpE* | CGGTTCTGTGGAGCGGTTT | TGAGCTTCTTCCGGATCAATG |
| *RpoA* | CGCATCAATTTGCGTCAAAG | GTTAGCTATAGGTTGTGCCGTATCAA |
| *RpoB* | CAAGTTTTCGGAGCCGAGAT | GCTAAAGATCCAGTAAGTCCAACGT |
| *RpoC1* | TCCGTCGGAACAACAATCTTG | TCCACGGCTTCTTGTACCAAT |
| *RpoC2* | ATGCATCGCAGGTACACCAA | CCCTCGCGTAAATTGCTTTG |
| *16S rRNA* | CCGTTGGTGTTCTTTCCGAT | TTCAAGTCCGCCGTCAAATC |
| *23S rRNA* | TGTGGGCGTTAGAGCATTGAG | CACTTGGCTACCCAGCGTTTA |
| *rpl21* | AAGAAGAGGAGGCTGCGGT | GACATTGGCGCCTTTCAGC |
| *rps7* | GCCAAAATCCATTCCAATTC | GGAGATGTACACGAGGAGATTG |
| *rps10* | CTGCCAACCAAGCGAAGAGT | ATCAATCGCTGGTGCGTTCT |
| *rps16* | CCTCGCGACAGACGTCCTAT | CTCCTCGTTAGGTGCTCCATC |
| *rps18* | CAACCTTTTCGCAAACCCAA | ATAATCAATTCGATCCCCCG |
| *rps20* | CACGCTCTTCTCCCTCTCCT | GTAGGAGGCGGACAGGCG |
| *UBQ5* | CTCGCCGACTACAACATCCA | TCTTGGGCTTGGTGTACGTCTT |

Additional file 1: Table S3: Vector construction primers

| Marker | Primer (5′-3′) | Restriction enzyme |
| --- | --- | --- |
| 930COMF | gctcggtacccggggatccGCCGCCATGTGTTCGCTC | BamHI |
| 930COMR | aggtcgactctagaggatccATAAAAAATACTCCTACTCTGCATTCAGA |  |
| WGL2-GFPF | caagacccttcctctaATGGCGCTCTCCCTCACCAC | SalI: |
| WGL2-GFPR | agcttgccgtaggtgACGCTTTGAGAACTGCGGGC |  |
| gR2tF-WGL2 | gcaGTGAAGCAGCGGCTTCCCGG gttttagagctagaaatagcaagttaaaataag | PstI |
| tR1tR-WGL2 | CCGGGAAGCCGCTGCTTCAC TGCACCAGCCGGGAATCGAAC | ApaI |

Additional file 1: Table S4: Effect of mutations in *cas* on WGL2

| Independent transgenic line | Site of mutation in genomic DNA | Mutation | Mutation of protein |
| --- | --- | --- | --- |
| *cas1* | 265 bp from ATG | 3-bp deletion | 89^th^ aa deletion |
| *cas2* | 270 bp from ATG | 1-bp insertion | 90^th^ aa frame-shift and premature translational termination at 205^th^ aa |
| *cas3* | 270 bp from ATG | 1-bp insertion | 90^th^ aa frame-shift and premature translational termination at 205^th^ aa |
| *cas4* | 271 bp from ATG | 1-bp insertion | 91^th^ aa frame-shift and premature translational termination at 205^th^ aa |
| *cas5* | 271 bp from ATG | 3-bp insertion | 91^th^ aa insertion |

Additional file 1: Table S5: Predicted genes in the mapped region

| Gene name | Function prediction |
| --- | --- |
| LOC_Os03g55830 | BTBN10 - Bric-a-Brac, Tramtrack, Broad Complex BTB domain with non-phototropic hypocotyl 3 NPH3 and coiled-coil domains, expressed |
| LOC_Os03g55840 | pentatricopeptide, putative, expressed |
| LOC_Os03g55850 | cold acclimation protein WCOR413, putative, expressed |
| LOC_Os03g55860 | retrotransposon protein, putative, unclassified, expressed |
| LOC_Os03g55870 | membrane associated DUF588 domain containing protein, putative, expressed |
| LOC_Os03g55874 | ATP synthase subunit beta, putative, expressed |
| LOC_Os03g55880 | expressed protein |
| LOC_Os03g55890 | ternary complex factor MIP1, putative, expressed |
| LOC_Os03g55920 | hydrolase, acting on carbon-nitrogen, putative, expressed |
| LOC_Os03g55930 | ribosomal protein, putative, expressed |
| LOC_Os03g55940 | expressed protein |
| LOC_Os03g55950 | H-BTB1 - Bric-a-Brac, Tramtrack, Broad Complex BTB domain with H family conserved sequence, expressed |
| LOC_Os03g55960 | EF hand family protein, putative, expressed |
| LOC_Os03g55970 | uncharacterized protein ycf44, putative, expressed |
| LOC_Os03g55980 | expressed protein |
| LOC_Os03g55990 | homeobox domain containing protein, expressed |
| LOC_Os03g56000 | PHLOEM 2-LIKE A10, putative, expressed |
